# Evidence based models of care for the treatment of alcohol use disorder in primary health care settings: a systematic review

**DOI:** 10.1186/s12875-020-01288-6

**Published:** 2020-12-05

**Authors:** Susan A. Rombouts, James H. Conigrave, Richard Saitz, Eva Louie, Paul Haber, Kirsten C. Morley

**Affiliations:** 1 Discipline of Addiction Medicine, Central Clinical School, Sydney Medical School, Faculty of Medicine and Health, The University of Sydney, Sydney, NSW 2006 Australia; 2 Centre of Research Excellence in Indigenous Health and Alcohol, Central Clinical School, Faculty of Medicine and Health, The University of Sydney, Sydney, NSW 2006 Australia; 3 Community Health Sciences, School of Public Health, Boston University, Boston, MA USA; 4 Drug Health Services, Royal Prince Alfred Hospital, Camperdown, NSW Australia

**Keywords:** Alcohol use disorder, Treatment, Primary health care, General practice, Pharmacotherapy

## Abstract

**Background:**

Pharmacological and behavioural treatments for alcohol use disorders (AUDs) are effective but the uptake is limited. Primary care could be a key setting for identification and continuous care for AUD due to accessibility, low cost and acceptability to patients.

We aimed to synthesise the literature regarding differential models of care for the management of AUD in primary health care settings.

**Methods:**

We conducted a systematic review of articles published worldwide (1998-present) using the following databases; Medline, PsycINFO, Cochrane database of systematic reviews, Cochrane Central Register of Controlled Trials and Embase. The Grey Matters Tool guided the grey literature search. We selected randomised controlled trials evaluating the effectiveness of a primary care model in the management of AUD. Two researchers independently assessed and then reached agreement on the included studies. We used the Cochrane risk of bias tool 2.0 for the critical appraisal.

**Results:**

Eleven studies (4186 participants) were included. We categorised the studies into ‘lower’ versus ‘higher’ intensity given the varying intensity of clinical care evaluated across the studies. Significant differences in treatment uptake were reported by most studies. The uptake of AUD medication was reported in 5 out of 6 studies that offered AUD medication. Three studies reported a significantly higher uptake of AUD medication in the intervention group. A significant reduction in alcohol use was reported in two out of the five studies with lower intensity of care, and three out of six studies with higher intensity of care.

**Conclusion:**

Our results suggest that models of care in primary care settings can increase treatment uptake (e.g. psychosocial and/or pharmacotherapy) although results for alcohol-related outcomes were mixed. More research is required to determine which specific patient groups are suitable for AUD treatment in primary health care settings and to identify which models and components are most effective.

**Trial Registration:**

PROSPERO: CRD42019120293.

## Background

Alcohol use disorder (AUD) is highly prevalent and contributes to 4% of the global disease burden and 5.3% of mortality worldwide [1]. Effective and safe treatments are available but are underutilised [2, 3]. For example, it is estimated that only 3% of AUD patients receive approved pharmacotherapy in Australia [4, 5]. In the USA, only 2.1% of a cohort with AUD were found to have been prescribed alcohol pharmacotherapy [6]. Moreover, time between onset of the disorder and initial treatment can be decades [2, 7].

Only 1 in 10 individuals with AUD perceive a need for treatment which possibly contributes to the low rate of enrolment and high dropout in specialty care [8]. Patients that do specifically seek AUD treatment are likely to be those with severe conditions, including greater alcohol intake and concurrent mental and physical comorbidity [3]. However, a significant proportion of AUD patients access primary health care, albeit for other reasons [9], and this represents an opportunity for earlier intervention. Primary health care appears to be an ideal treatment setting for AUD due to this accessibility but also due to low costs and acceptability for patients.

Primary care settings are able to provide longitudinal, comprehensive and coordinated care with medication management [10]. Patients commonly present to primary care for problems related to AUD such as mood disorders, hypertension, injuries and others. The chronic and relapsing nature of some with AUD make this type of care appropriate and necessary. Indeed, while the rate of prescribing of AUD pharmacotherapy is low, one recent study demonstrated that clients who had more contact with the primary care system were more likely to be prescribed AUD medications [6]. Identifying and treating early-stage AUD in these settings can potentially prevent conditions deteriorating.

In recent years, several models of care have been evaluated in primary care settings. The ‘screening, brief intervention and referral to specialty care (SBIRT)’ model is best known and multiple systematic reviews confirm its effectiveness [11–13]. However, in the management of moderate-severe AUD, the effectiveness of SBIRT is limited at best [3, 14, 15]. Integrated models of care or pathways have been developed, whereby the treatment is delivered either by the general practitioner or an on-site nurse practitioner.

Accordingly, we aimed to synthesise the existing models of care, other than SBIRT, for the management of AUD in primary care settings. We sought to evaluate the effectiveness of these care models with regards to increasing treatment engagement (e.g. number of visits and/or uptake of AUD pharmacotherapy) and reducing alcohol consumption and to provide recommendations for further research.

## Methods

We followed the Preferred Reporting Items for Systematic Reviews and Meta-analyses (PRISMA) guidelines for systematic reviews [16]. We registered the systematic review with the international Prospective Register of Systematic reviews (PROSPERO: CRD42019120293). Additional information on the methods can be found in the published protocol [17].

### Eligibility criteria

Studies were eligible if: 1) they were published in English, 2) they were published after 1 January 1998 (to allow for a 20 year period from search commencement), 3) they compared models for the management of AUD, and 4) at least 80% or more of the subjects had an AUD, or if results for subjects with AUD were presented separately to those with other conditions. We excluded languages other than English given the costs and time required for translation were unavailable.

Our interventions of interest are complex health interventions which target how care is organised in addition to types of treatments. For inclusion, the model of care had to cover several parts of the care pathway other than screening. The setting had to be in primary health care using primary care physicians, nurse practitioners and/or case managers. Consultations with specialty care was accepted. Treatment facilities had to be physically in or attached to the primary care clinic. We excluded studies where the independent variable was the specific treatment rather than the model of care. We also excluded articles examining SBIRT (screening, brief intervention, referral to treatment) for individuals with mild AUD unless a novel component was added to the model of care.

### Search strategy

We searched Medline, PsycINFO, Cochrane database of systematic reviews, Cochrane Central Register of Controlled Trials (CENTRAL) and Embase (2019). We conducted reference searches of relevant reviews and articles. Grey Matters tool, which is a checklist of health-related sites organized by topic, and Google were used in the grey literature search. Authors of identified conference abstracts were contacted for additional information about their study and potential availability of preliminary data. Before publication of this systematic review we ran the search again to include all newly published studies (04/06/2020).

See Appendix 1 for our search strategy in Medline and Appendix 2 for grey literature.

### Study selection

Initially, duplicates were removed from the database after which all the titles were screened with the purpose of discarding irrelevant articles (unrelated to alcohol treatment or primary care). The remaining papers were included in an abstract and full-text screen. All steps were completed by one researcher (SR) with consultation with two other researchers (KM and JC). Disagreements were resolved in consensus-based discussion.

### Data extraction and synthesis

Key information extracted from the articles included design of the study; type of participants; study setting; type of intervention/ model of care; type of health care worker; duration of follow-up and outcome measures. Outcome data on treatment engagement (e.g. number of visits and/or uptake of AUD pharmacotherapy or any treatment) and alcohol use were extracted. Categorical outcomes were converted into log odds ratios (OR) and log incidence rate ratios (IRR). Continuous measures were converted into standardized mean differences (SMD). Data extraction was completed by one researcher (SR) with error checking by two other researchers (JC and KM). Due to variability in study design, measures and outcome data reporting, we were unable to extract sufficient data to perform a meta-analytic synthesis.

### Quality appraisal

All studies were critically assessed by two researchers independently using the Revised Cochrane risk-of-bias tool (RoB 2.0) [18]. Meta-biases such as outcome reporting bias was evaluated by determining whether the protocol was published before recruitment of patients. Additionally, trial registries were checked to determine whether the reported outcome measures and statistical methods matched original protocols. We also reported on funding from the pharmaceutical industry. To minimise publication bias, we looked at conference abstracts and grey literature.

## Results

**Fig. 1:**
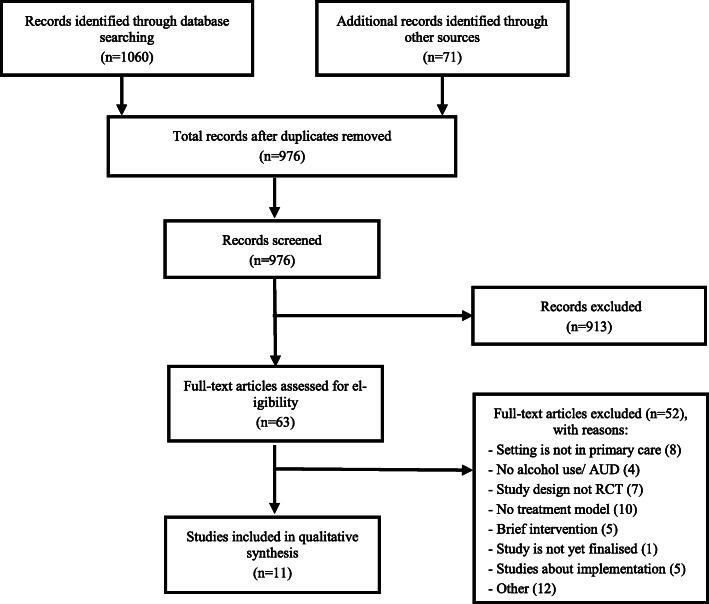
Flow diagram of the study selection process

The literature search including synonyms for ‘model of care’ returned 1060 records. An additional 71 records were identified from other sources (Fig. 1). The details of the included studies (*n* = 11) are summarised in Table 1 according to intensity and/or duration of care (from low to high).

**Table 1:** Study characteristics

Study, year	Design, study duration	Setting (country; type of health care professional)	Participants (inclusion criteria + recruitment details)	Intervention
*Low intensity models of care*				
Moore et al., 2010 [19]	RCT 12 months	United States Community based PC clinics	≥55 years, at risk drinkers identified by the CARET (*n* = 631) Recruitment: in primary care (not seeking treatment for AUD)	**Multifaceted intervention:** Personalized patient reports (educational booklet; a drinking diary). Drinking risk reports for physicians to guide drinking discussion. Telephone behavioural counselling 3x (at 2, 4, 8 weeks) **Control group:** Usual PC + a booklet outlining recommended behaviours for alcohol use, nutrition, exercise, medication use and smoking.
Ettner et al., 2014 [20]	RCT (cluster) 12 months	United States Community based PC clinics	≥60 years, at risk drinkers identified by the CARET (*n* = 1186) Recruitment: in primary care (not seeking treatment for AUD)	**Educational intervention:** Emailed personalized patient report (educational booklet; a drinking diary; 13 tips sheets) at baseline and 6 months. Drinking risk reports for physicians about patients to guide drinking discussion, handed to physician before every scheduled visit. Telephone behavioural counselling 3x (at baseline, 3-months and 6 months) **Control group:** Usual PC, which could have included alcohol counselling
Wallhed Finn et al., 2018 [21]	RCT 6 months	Sweden Community based PC clinic	≥ 18 years, Alcohol dependence according to ICD-10. (*n* = 288) Recruitment: in primary care (not seeking treatment for AUD) + via advertisement in newspapers (seeking treatment for AUD)	**15-method (stepped-care):** Various steps conducted by general physician. Step 1: identification of problem drinking and brief advice; Step 2: Assessment + 30-min feedback; Step 3: 4 sessions based (15 min) on CBT and MET. *Sessions can be combined with pharmacological treatment (acamprosate, disulfiram, nalmefene, or naltrexone) *Referral to next step happened when patient score > 15 points on the AUDIT **Control group:** Specialist treatment. Same pharmacological treatment was offered as in the intervention. Various options of psychological treatment (4 to 12 sessions of 45 min)
Drummond et al., 2009 [22]	RCT 6 months	United Kingdom Community based PC clinics	Men, age ≥ 18 years, AUDIT ≥8 and/or diagnosis of AUD using ICD-10 criteria and/or > 21 SD/week or > 8 SD/day (*n* = 112) Recruitment: in primary care (not seeking treatment for AUD)	**Stepped care interventions:** Step 1: 40 min session of behavioural change counselling; Step 2: MET (max four 50 min sessions on weekly basis); Step 3: referral to specialist alcohol treatment. *Referral to next step happened when patients still consumed alcohol at hazardous levels after 4 weeks **Control group:** 5-min structured brief intervention + short self-help booklet outlining consequences of excessive alcohol consumption.
Coulton et al., 2017 [23]	RCT 12 months	United Kingdom Community based PC clinic	≥ 55 years, AUDIT ≥8 (*n* = 529) Recruitment: in primary care (not seeking treatment for AUD)	**Stepped-care interventions**: Step 1: 20 min session of behavioural change counselling; Step 2: MET (three 40 min sessions on weekly basis) Step 3: referral to specialist alcohol treatment. *Referral to next step happened when patients still consumed alcohol at hazardous levels after 4 weeks **Control group:** 5-min structured brief intervention + short self-help booklet outlining consequences of excessive alcohol consumption.
*High intensity models of care*				
Oslin et al., 2013 [24]	RCT 6.5 months	United States VA primary care clinics	≥18 years, DSM-IV criteria for current alcohol dependence, and > 2 SD/ day for 60 days prior to randomization. (*n* = 163) Recruitment: in primary care (not seeking treatment for AUD) + patient request (seeking treatment for AUD)	**Alcohol care management:** Weekly 30 min visits with BHP (assess alcohol use, encouraged treatment adherence, offered support and education, monitoring medical problems, education about pharmacotherapy). Promotion of evidence-based pharmacotherapy (naltrexone 50 mg), however use was not a requirement of participation. As participants improved, the frequency of visits could be reduced to twice per month after the first 3 months. **Control group:** Standard specialty care at the VA specialty outpatient addiction program, based on the 12-step facilitation model, including assessments, outpatient detoxification, counselling, pharmacotherapy, psychotherapy, psycho-educational groups, outreach and referral, and acupuncture. Patients were to be expected to attend Alcoholics and Anonymous.
Watkins et al., 2017 [25] (SUMMIT-trial)	RCT 6 months	United States Federally qualified health center (primary care)	≥ 18 years, probable OAUD according to ASSIST. (*n* = 377) Recruitment: in primary care (not seeking treatment for AUD)	**Collaborative care:** 6 sessions brief psychotherapy treatment and/or medication-assisted treatment. On-site behavioural health care, integration of addiction expertise through clinical psychologist with motivational interviewing experience, first appointment with care coordinators, entry into registry to track treatment progress and to prompt care coordinators to reach out to patients with missed appointments. **Control group:** Usual PC; participants were told that the clinic provided OAUD treatment and given a number for appointment scheduling and list of community referrals.
Upshur et al., 2015 [26] Project RENEWAL	RCT 6 months	United States Health care for the homeless clinic	≥ 18 years women seeking primary care services who screened positive for hazardous drinking (AUDIT-C score > 4) (*n* = 82) Recruitment: in primary care (not seeking treatment for AUD)	**Chronic care model:** First, participants would get a brief intervention from the PCP and referral to the Care manager (CM) for ongoing care. PCP would provide 4–6 appointments for ongoing care and encouragement of addiction medication. The CM was asked to complete at least 15 phone or in-person follow-up sessions in the 6 months. **Control group:** Usual PC + access to the specialty care offered in the clinic (e.g. counselling, psychiatry, etc).
Bradley et al., 2018 [27] (CHOICE-trial)	RCT (encouragement); 12 months	United States VA primary care clinics	Age 21–75; heavy drinking (≥4 SD/occasion for women; ≥5 SD for men) at least twice per week or once per week if prior alcohol treatment (*n* = 304) Recruitment: in primary care (not seeking treatment for AUD)	**Alcohol care management:** 1–2 engagement visits (focus on life goals, feedback from baseline assessment, using MET/SDM). Repeated nurse visits (review patient self-monitoring and/or biomarker) + provide behavioural goal setting skills development for reducing drinking, AUD medications, withdrawal management, mutual help, and referral to specialty addictions treatment per patient preference. **Control group:** Usual PC (offered annual behavioural health screening, integrated mental health services, and access to specialty mental health and addictions clinics)
Saitz et al., 2013 [28] (alcohol subgroup) (AHEAD-trial)	RCT 12 months	United States Hospital based PC clinic	≥ 18 years, Alcohol dependence according to CIDI-SF and heavy drinking in the 30 days (≥5 SD/occasion at least twice or ≥ 22 drinks per week in an average week; ≥4 and ≥ 15, respectively, for women) (*n* = 409). Recruitment: detoxification facility, referrals from hospital and advertisements (treatment seeking for AUD)	**Chronic care management:** Study clinic with multidisciplinary team located in PC. Two 90-min visits separated by 3–4 days receiving assessments by all 4 clinicians. Four sessions of MET, relapse prevention, pharmacotherapy was offered as appropriate, facilitated referrals to addiction specialty care, drop in care and 24 h pager access. **Control group:** PC + a list of addiction treatment resources. They were given a phone number to access 4 MET sessions.
Willenbring et al., 1999 [29]	RCT 24 months	United States Outpatient clinic- Minneapolis VA medical center (MVAMC)	Patients with current diagnosis of severe medical illness due to alcohol use (e.g. alcoholic liver disease, alcoholic pancreatitis, etc.), recent pathological drinking (past 6 months) (*n* = 105) Recruitment: referral by medical providers + patients were identified when presenting to acute treatment units (not seeking treatment for AUD)	**Integrated outpatient treatment:** Primary care professionals are principal caregiver. First, patient receive 1–2 day inpatient evaluation by a multidisciplinary team (internist, psychiatrist, nurse practitioner, psychologist, social worker) who make a treatment plan. After which, they are seen monthly for assessment and feedback (e.g. biological indicators) and offer of a support group. Important facets of the care provided are: case management, aggressive follow-up, and family involvement. **Control group:** Standard specialty care: separate referrals for alcohol treatment and outpatient primary medical care. Alcoholism counsellors/ mental health professionals are principal caregiver in the alcoholism treatment.

*CARET* comorbid alcohol risk evaluation tool, *AUDIT* alcohol use disorder identification test, *CIDI-SF* Composite International Diagnostic Interview-Short Form, *ASSIST* Alcohol, smoking and substance involvement screening test, *ICD-10* International Statistical Classification of Diseases (10th revision), *DSM* Diagnostic and Statistical Manual of Mental Disorders, *OAUD* Opioid and alcohol use disorders, *GDO* Good drinking outcome;

* Statistically significant *P* < 0.05

### Population

This systematic review included 11 studies with a combined number of 4186 participants (72% male). Identification of hazardous alcohol use or AUD differed among the studies, ranging from utilizing assessment tools to more formal diagnosis of AUD using the International Statistical Classification of Diseases (10th revision) (ICD-10) or according to the Diagnostic and Statistical Manual of Mental Disorders (DSM-IV) criteria for current alcohol dependence*.* The study by Willenbring et al. only included participants with a current diagnosis of severe medical illness due to excessive alcohol use (e.g. alcoholic pancreatitis) [29]. Three studies had strict exclusion criteria regarding current substance abuse and dependence [21, 22, 24] other than alcohol (a maladaptive pattern of substance use, such as cannabis or amphetamines, leading to clinically significant impairment and distress) while others did not mention this exclusion criterion. Two studies specifically included patients with AUD and substance use disorder (SUD) [25, 28]. Watkins et al. reported that 94% of the sample had an AUD, of which 40% had both an opioid and alcohol use disorder (OAUD) [30]. Data for the AUD subgroup without comorbid opioid dependence was obtained from the authors upon request. The study by Upshur et al. specifically included homeless women with AUD, whilst most others excluded homeless people from their studies [26].

### Setting

Two studies were conducted in the United Kingdom [22, 23], one study was set in Sweden [21] and the remaining eight trials in the United States. Most studies were conducted in community primary care settings [19–23] and there were three studies set in VA primary care clinics [24, 27, 29]*.* Other locations included a hospital-based primary care clinic [28]*,* a health centre for the homeless [26] and a federally qualified health centre [25]*.*

### Study-design

Two studies were cluster-randomised controlled trials [20, 26] and one study was labelled a randomised encouragement trial that offered services to patients but did not require that they accept [27]*.* None of the studies blinded participants or physicians. Six studies included blinded assessment of the outcomes (researchers were unaware of the patients’ group assignment) [19, 20, 22, 24, 25, 27], however, alcohol consumption was often obtained by self-report (e.g. standard drinks (SD) of alcohol per week).

### Intervention

The models of care in each of the studies differed significantly with regards to the duration, the setting, health professionals engaged and access to types of treatment. As a result, following data extraction, we decided to divide the models of care into lower intensity models and higher intensity models whereby the components for each of these are depicted in detail in Table 2.

**Table 2:** Components of model of care

	Moore 2010 [19]	Ettner 2014 [20]	Wallhed-Finn 2018 [21]	Drummond 2009 [22]	Coulton 2017 [23]	Oslin 2013 [24]	Watkins 2017 [25]	Upshur 2015 [26]	Bradley 2018 [27]	Saitz 2013 [28]	Willenbring 1999 [29]
	*Lower intensity models*					*Higher intensity models (longitudinal care)*					
Identification											
Screening	X	X	X	X	X	X	X	X	X	X	X
(E) MR alterations	X	X					X	X	X	X	
Increasing patient engagement											
Follow-up (active)							X		X	X	X
Shared-decision making			X				X	X	X	X	
Goal setting (flexibility)			X			X		X	X		X
Self-management support						X	X	X	X	X	
Patient education (material)	X	X				X		X	X		
Biomarker feedback									X	X	X
Support system involvement											X
Education health professionals											
Training of staff (> 1 h)			X	X	X	X	X	X	X	X	
Feedback/ supervision					X	X	X	X	X		
Specialist/ expert consultations					X		X	X	X	X	X
Staff											
Primary care staff**	X	X	X	X	X	X	X	X	X	X*	X
Psychologist/ alcohol therapist				X	X	X	X	+		X	
Medical specialist							X	+		X	X
Case manager/ care coordinator							X	X	X	X	X
Treatment											
(Brief) psychosocial therapy	X	X	X	X	X	X	X	X	X	X	X
AUD pharmacotherapy			X			X	X	X	X	X	
Self-help groups							X	X	X	X	X
Linkage to specialty services											
Referral to specialty care				X	X		X	X	X	X	X
Social/community services							X	X		X	X
Duration of intervention (treatment)											
Up to three months	X		X	X	X						
Three to six months						X	X	X			
Six to twelve months		X							X	X	
Up to 24 months											X

Lower intensity models = e.g. extended brief intervention, stepped care intervention); Higher intensity intervention/longitudinal care plan = e.g. chronic care model, collaborative care model, alcohol care management; (E)MR = (electronic) medical records; AUD = alcohol use disorder;(Brief) psychosocial therapy varies from counselling, motivational enhancement therapy to cognitive behavioural therapy

* intervention staff was a multidisciplinary team separate from any primary care staff but patients in the intervention group do receive a primary care appointment

** Primary care staff (general physician, nurse (practitioner), health educator)

+ both intervention and control group had unrestricted access to specialist care offered in the clinic (e.g. counselling, psychiatry, dental services, vision services, etc.) – not specific to the model

#### Lower intensity models

The studies by Moore et al. [19] and Ettner et al. [20] evaluated a multi-faceted model with personalised patient reports, educational booklets and a drinking diary to educate patients about their drinking habits. The primary care physician would also receive a drinking report prior to every scheduled appointment to stimulate discussion about alcohol consumption. Subsequently, patients would receive 3 telephone behavioural counselling sessions. These two studies differed with regards to the timing of these counselling sessions (frontloading versus more spread out, respectively). The health professionals included a primary care physician and health educators.

The studies by Wallhed-Finn et al. [21], Drummond et al. [22] and Coulton et al. [23] evaluated a variation of a stepped-care model. They all started with a standard brief intervention (5–10 min). The intensity of the treatment increased when patients continued to drink at hazardous levels. Treatment included feedback, behavioural counselling (based on cognitive behavioural therapy (CBT) and/or motivational enhancement therapy (MET). Referral to specialty care would be followed if necessary. The model evaluated by Wallhed-Finn et al. [21] was unique in that it provided psychosocial therapy adapted to the context and time constraints of primary care with the option for any pharmacological treatment.

#### Higher intensity models (longitudinal care models)

Six of the included studies [24–29] assessed the effectiveness of models of care that were based on elements of the collaborative care/ chronic care model (CCM) [31, 32]. The six studies offered high intensity intervention with psychosocial support (MET and/or CBT) and pharmacological treatment for AUD. They all integrated addiction expertise and behavioural counselling support and assured good communication between primary care physicians and other health professionals using the electronic medical system (EMR). Often a case manager kept track of treatment and attendance, assuring active follow-up. To increase treatment engagement, CCM concepts such as shared-decision making and self-management support were incorporated in these studies. Shared decision making directed the duration, length, type and intensity of the treatment. Self-management support was usually provided by biomarker testing feedback and routine assessment. Two out of six studies utilised specialty addiction treatment as the comparator. The remaining studies compared the care model against usual primary care with access to specialty addiction treatment resources.

#### Control groups

Control groups are described in detail in Table 1. These included usual primary care plus possible addition of alcohol counselling [20], an education booklet [19], 5 min structured brief intervention with self-help booklet [22, 23], provision of a number for outpatient treatment [25, 28], specialty counselling or psychiatry [26], annual behavioural health screening and integrated mental health services [27]. Addiction specialty treatment was the comparator model of care in three studies but may have been provided separately [21, 24, 29].

### Quality appraisal

Overall, the quality of the studies was mixed with most trials having a moderate risk of bias for both engagement and drinking outcome measures (see Table 3). More specifically, the majority of studies had low risk of bias arising from the randomisation process except for some risk of bias regarding cluster randomisation with Ettner et al. [20] and Upshur et al. 2015. With regards to bias due to deviations from the intended intervention in terms of assignment to intervention, the majority of studies had low risk of bias although our appraisal yielded some-high bias for Oslin et al. [24] and high bias for the Willenbring et al. [29]. All the studies were judged to have low risk of bias in terms of adhering to the intervention. Bias with regards to missing outcome data was observed in several studies including Drummond et al. [22], Watkins et al. [25], Willenbring et al. [29] and Upshur et al. [26]. Bias with regards to measurement of outcome was observed to some degree in all the studies except for Ettner et al. [20]. Half of the studies were judged to have some risk of bias regarding selection of reported results. Funding from the pharmaceutical industry was not apparent in 10 of the studies. In one of the studies, Watkins et al. [25], Alkermes provided long-acting injectable naltrexone at no charge to patients. None of the studies were blinded and all studies used self-reported measures for alcohol consumption.

**Table 3:** Bias assessment of engagement outcome measures (first) and clinical/drinking outcome measures (second)

Reference	Domain 1; bias arising from the randomization process	Domain 1b; bias arising from the randomization process (cluster-randomized trials)	Domain 2; bias due to deviations from the intended intervention (assignment to intervention)	Domain 2; bias due to deviations from the intended interventions (adhering to intervention)	Domain 3; bias due to missing outcome data	Domain 4; bias in measurement of the outcome	Domain 5; bias in selection of the reported results	Overall risk of bias judgement
Bradley et al., 2018	Low risk Low risk	NA	Low risk Low risk	Low risk Low risk	Low risk Low risk	Low risk Some risk	Some risk Some risk	Some risk Some risk
Coulton et al., 2017 [23]	NA Low risk	NA	NA Low risk	NA Low risk	NA Low risk	NA Some risk	NA Low risk	NA Low risk
Drummond et al., 2009 [22]	NA Low risk	NA	NA Low risk	NA Low risk	NA High risk	NA Some risk	NA Some risk	NA High risk
Ettner et al., 2014 [20]	Low risk Low risk	Some risk Some risk	Low risk Low risk	NA NA	Low risk Low risk	Low risk Low risk	Low risk Low risk	Some risk Some risk
Moore et al., 2010 [19]	NA Low risk	NA	NA Low risk	NA Low risk	NA Low risk	NA Some risk	NA Some risk	NA Some risk
Oslin et al., 2013 [24]	Low risk Low risk	NA	Some risk High risk	Low risk Low risk	Low risk Low risk	High risk Low risk	Some risk Some risk	High risk High risk
Saitz et al., 2013 [28]	Low risk Low risk	NA	Low risk Low risk	Low risk Low risk	Low risk Low risk	Some risk Some risk	Some risk Some risk	Some risk Some risk
Watkins et al., 2017 [25]	Low risk Low risk	NA	Low risk Low risk	Low risk Low risk	Low risk Some risk	Low risk Some risk	Low risk Low risk	Low risk Some risk
Willenbring et al., 1999 [29]	Low risk Low risk	NA	High risk High risk	Low risk Low risk	Some risk Some risk	Low risk Some risk	Some risk Some risk	High risk High risk
Wallhed finn et al., 2018 [21]	Low risk Low risk	NA	Low risk Low risk	Low risk Low risk	Low risk Low risk	Low risk Some risk	Low risk Low risk	Low risk Low risk
Upshur et al., 2015 [26]	Low risk Low risk	Some risk Some risk	Some risk Some risk	NA NA	Some risk Low risk	Some risk Low risk	Some risk Some risk	High risk High risk

### Effectiveness

We aimed to evaluate effectiveness of models of care in primary care-settings in increasing treatment engagement and reducing alcohol consumption via meta-analytic synthesis. However, due to the small number of studies, high heterogeneity between studies, and due to large variations in outcome measures, meta-analysis was not feasible. We thus illustrated patterns using tables.

#### Treatment engagement

We tabulated treatment engagement outcomes with significant results (Table 4). There was a high heterogeneity between studies in outcome measures for treatment engagement. The uptake of AUD medication was reported in 5 out of 6 studies that offered AUD medication. Three studies reported a significantly higher uptake of AUD medication in the intervention group.

**Table 4:** Engagement measures

Reference, study duration	Outcome measures	Results (95% Confidence interval)
Moore et al. 2010 [19] 12 months	NA	
Ettner et al. 2014 [20] 12 months	-Alcohol discussion with PC physician, %	I = 23% vs C = 13%**
Wallhed-Finn et al. 2018 [21] 6 months	-Number of visits, mean	I = 2.9 vs C = 4.7***
	-Duration of treatment, min	I = 74 vs C = 187***
	-AUD pharmacotherapy, %	NS
Drummond et al. 2009 [22] 6 months	NA	
Coulton et al. 2017 [23] 12 months	NA	
Oslin et al. 2013 [24] 6.5 months	-Mean number of visits (SD)	NS
	-Proportion of patients with at least two addiction treatment visits	OR 6.97 (4.04, 12.05)***
	-Patients treated with naltrexone, %	I = 65.9 vs C = 11.5***
Watkins et al. 2017 [25] SUMMIT trial (Data of AUD subgroup without comorbid opioid dependence) 6 months	-Patients received any evidence-based treatment, %	I = 39.4 vs C = 15.2; OR 5.09 (2.33–11.14)***
	-Patients received any brief treatment, %	I = 37.5 vs C = 10.10; OR 7.70 (3.33–18.32)***
	-Patients received any medication assisted treatment, %	NS
	-HEDIS initiation, %	I = 32.69 vs C = 9.09; OR 6.16 (2.56–14.85)***
	-HEDIS engagement, %	I = 14.42 vs C = 4.04; OR 6.56 (1.78–24.15)*
Upshur et al. 2015 [26] Project RENEWAL 6 months	-Number of visits, mean (6 mo)	I = 12.1 vs C = 6.2**
	-Meet criteria for spending time in drug/alcohol treatment, % (3 &6 mo)	NS, NS
	-Talking about substance abuse with counsellor, % (3 & 6 mo)	3 mo: I = 67.6 vs C = 30.6** 6 mo: NS
	-Attending AA meetings, % (3 & 6 mo)	NS, NS
	-Patients visiting mental health provider, %, (3 & 6 mo)	NS, NS
	-Total contacts with any substance use service, % (3 & 6 mo)	3 mo: I = 75.7 vs C = 44.4* 6 mo: I = 75 vs C = 47.2**
Bradley et al. 2018 [27] CHOICE trial 12 months	-AUD medication use, % (3&12 mo)	3 mo: I = 14 vs C = 4.6** 12 mo: I = 32 vs C = 8.4***
	-AUD medication use> 30 days, %, (3 &12 mo)	3 mo: I = 9.3 vs C = 2.6** 12 mo: I = 26.0 vs C = 7.1***
	-VA addictions treatment, %, (3&12 mo)	NS, NS
	-AA involvement, % (12 mo)	NS
	-Any alcohol-related care, % (3&12 mo)	3 mo: I = 18 vs C = 8.4** 12 mo: I = 42.0 vs C = 26.0**
Saitz et al. 2013 [28] AHEAD trial (Data of AUD subgroup with/ without SUD) 12 months	-Any mutual help meeting attendance, %	NS
	-Any addiction treatment, %	I = 43 vs C = 42; OR 1.36 (1.01–1.84)*
	-Any inpatient addiction treatment, %	NS
	-Any addiction medication, %	I = 16 vs C = 10; OR 2.12 (1.29–3.48)**
Willenbring et al. 1999 [29] 24 months	-VA hospital days over prior 2 year, psychiatric and alcohol treatment, mean	NS
	-VA clinic visits over prior 2 years	I = 42.2 vs C = 17.4**

*Mo* months, *VA* Veterans Affairs, *NS* not significant; *p < 0.05, **P < 0.01; ***P < 0.001. Shading: indicates addiction specialty care as control group

#### Reduction of alcohol consumption

Clinical outcomes relating to alcohol consumption are presented in Table 5. Similarly, there was a high heterogeneity between the clinical outcome measures. Significant reductions in alcohol consumption in patients treated in primary care settings relative to comparison groups were reported in almost half of the studies (two out of five lower intensity models; three out six higher intensity models). The studies by Bradley et al., Saitz et al. and Upshur et al. reported alcohol reduction in both the intervention and control group.

**Table 5:** Clinical outcomes / alcohol consumption measures

Reference	Outcome measures	Results (95% Confidence Interval)
Moore et al. 2010 [19] 12 months	-At-risk drinking, % (3 & 12 mo)	3 mo: I = 49.6 vs C = 61.2; OR 0.41 (0.22–0.75)** 12 mo: NS
	-One or more HDD in past 7 days, % (3 & 12 mo)	3 mo: I = 10.3 vs C = 16.9; OR 0.46 (0.22–0.99)* 12 mo: NS
	-Number of drinks in past 7 days, mean (3 & 12 mo)	3 mo: I = 8.9 vs C = 10.7; OR 0.79 (0.7–0.9)*** 12 mo: I = 9.39 vs C = 10.70; OR 0.87 (0.76–0.99)*
Ettner et al. 2014 [20] 12 months	-At-risk dinking, % (6 & 12 Mo)	6 mo: I = 60 vs C = 72** 12 mo: I = 56 vs C = 67 **
	-Drinks per week, no, (6 & 12 Mo)	6 mo: I = 9.82 vs C = 12.24** 12 mo: I = 9.45 vs C = 11.64**
Wallhed-Finn et al. 2018 [21] 6 months	-Weekly alcohol consumption, gr	NS
	- Heavy drinking days per month	NS
	-ICD-10 criteria dependence at follow up	NS
	-SIP total score	NS
	-Proportion patients drinking under recommended levels	NS
Drummond et al. 2009 [22] 6 months	-Total number of drinks consumed in period	NS
	-Drinks per drinking day	NS
	-Percentage of days abstinent	NS
	-Alcohol problems questionnaire	NS
Coulton et al. 2017 [23] 12 months	-Average drinks per day, mean (6 & 12 mo)	NS, NS
	-AUDIT-C score, mean (6 &12 mo)	NS, NS
	-AUCIT-C score, positive %, (6 &12 mo)	NS, NS
Oslin et al. 2013 [24] 6.5 months	-Presence/absence heavy drinking	NS
	-Percent days heavy drinking^a^	OR 2.16 (1.27, 3.66)*
	-Presence/ absence of any drinking	NS
	-SIP	NS
Watkins et al. 2017 [25] SUMMIT trial (Participant data of AUD subgroup without comorbid opioid dependence) 6 months	-Abstinence from all opioids and any alcohol, past 30 days, %	I = 25.32 vs C = 15.71; β 0.21 (0.07–0.35)*
	-Abstinence from opioids, any alcohol, cocaine, methamphetamines and marijuana, past 30 days, %	I = 21.52 vs C = 14.29; β 0.17 (0.04–0.30)*
	-Heavy drinking, past 30 days, %	NS
	-Abstinence from all opioids and no heavy drinking, %	I = 44.29 vs C = 36.51; β 0.26 (0.10–0.42)**
	-SIP score, alcohol & drugs score, mean	NS
Upshur et al. 2015 [26] Project RENEWAL 6 months	-Reduction in of drinks per month (baseline to 6 months), median	I = 185 SD/month to 12 SD/month** C = 87.3 SD/month to 1.3 SD/month**, difference between I and C = NS
	-Nr drinks/month last 3 months, Median (SD) (3 & 6 mo)	NS, NS
	-Nr drinks last 3 months (3 & 6 mo)	NS, NS
	-Alcohol use consequences, mean (SD) (3 & 6 mo)	NS, NS
Bradley et al. 2018 [27] CHOICE trial 12 months	-Heavy drinking days, % (3 &12 mo)	NS, NS
	-Patients with good drinking outcomes, % (3 & 12 mo)	NS, NS
	-Patients with no heavy drinking days, % (3 &12 mo)	NS, NS
	-Days abstinent, % (3 & 12 mo)	3 mo: I = 30 vs C = 38* 12 mo: I = 35 vs C = 45*
	-Patients abstinent, % (3 &12 mo)	NS, NS
	-Patient drinking below weekly limits, %	NS, NS
	-SIP score, mean	NS, NS
Saitz et al. 2013 [28] AHEAD trial (Data of AUD subgroup with/ without SUD) 12 months	-Abstinence from heavy drinking, past 30 days, %	NS
	-No. of heavy drinking days in past 30 days, mean	NS
	-Alcohol-related problem score, mean	I = 10.4 vs C = 13.1; OR 0.85 (0.72–1.00)*
Willenbring et al. 1999 [29] 24 months	-Positive DSM-II-R criteria (0–9), No	NS
	-Drinking days during last 30 days, mean	I = 3.7 vs C = 7.0*
	-Drinks per drinking day, No	I = 1.8 vs C = 3.0*
	-Days since last drink, mean	NS
	-Abstinent, %	I = 74 vs C = 48*

*Mo* months, *NS* not significant, *OR* Odds ratio, *SD* standard drink; *p < 0.05, **P < 0.01; ***P < 0.001; ^a^ Timing of measurements unknown. Shading: indicates addiction specialty care as control group

Heavy drinks per drinking day (HDD) in the past month at follow-up was reported by four studies, three of which were considered higher intensity models of care. The definition of HDD most commonly used was: women ≥4 SD and men ≥5 SD per day of approximately 14 g of ethanol per SD. However, the study by Wallhed-Finn et al. considered HDD as: women > 3 SD and men> 4 SD of 12 g per day. Only the study by Oslin et al. reported a benefit of intervention compared to control for this outcome measure.

## Discussion

In the current review we examined the evidence base supporting treatment of AUD in primary care settings, providing an overview of the models of care. The models of care were generally aligned to either lower intensity models of care such as extended brief intervention and stepped care or higher intensity care models that were often based on the principles of the collaborative care/chronic care model (CCM) [10, 31–34]. We were unable to extract sufficient data to conduct a meta-analysis due to variability in study measures and outcome data reporting. Nonetheless, we observed that the majority of care models improved treatment engagement of AUD patients, although the lower intensity models often did not report engagement outcomes. Significant reductions in alcohol consumption in patients treated in primary care settings relative to comparison groups were reported in less than half of the studies (two out of five lower intensity models; three out six higher intensity models) with more than half (seven out of eleven studies) reporting significant reductions in any alcohol outcomes (e.g. heavy drinking or alcohol-related problems).

Several methodological differences may explain mixed findings with regards to alcohol outcomes, such as inconsistent treatment compliance, shorter treatment duration and inadequate training of staff and/or lack of fidelity measures for psychosocial techniques. In addition, negative studies all reported similar reductions of alcohol consumption in both the intervention and control group, which may indicate issues with study design regarding comparison groups. None of the studies were blinded and, for example, in the study by Upshur et al. feedback of screening was provided to all participants which may have served as a brief intervention, prompting physicians to commence AUD treatment [26] or for mild AUD patients [35] to reduce consumption [14, 36].

Regarding higher intensity models of care, there were three studies that reported significant reductions in alcohol consumption (reduced HDD or increased abstinence), relative to control [24, 25, 29, 30]. These studies did not include participants with co-morbid SUD, and for those that did, the beneficial results were restricted to the AUD participants only [24, 25, 29, 30]. In comparison, the higher intensity trials with null results included individuals with co-morbid SUD [26–28]. It is thus possible that the primary care model may be somewhat limited for patients with more complex needs, although studies with CCM for other conditions have reported effectiveness, even in patients with high social needs and co-morbidity [37]. Higher intensity models also often included patients with higher drinking levels and engagement of multiple healthcare professionals (e.g. psychologists, medical specialists, case managers). While the current systematic review demonstrates that provision of AUD treatment can be implemented in primary care, there is a gap in the evidence base regarding our capacity to define which patients are suitable for AUD treatment in primary care and which interventions are effective. Finally, the issue of feasibility in terms of time constraints and resources, particularly for complex patients, should not be underestimated as a barrier to widespread adoption of AUD treatment in primary care.

It is worth noting that our findings suggest pharmacotherapy can be simply and safely provided in the primary care setting. Which may lead to increased uptake and engagement with AUD treatment. There is thus potential for wide-spread benefit should primary care physicians adopt the responsibility for recognition, screening and prescribing. The provision of education regarding pharmacological treatment options could overcome some previously noted barriers such as lack of knowledge about the available treatment possibilities and misconceptions about medication efficacy [38–40].

### Recommendations for primary care alcohol treatment research

#### Alcohol outcome measures

Future alcohol treatment research in primary care settings will require more consistent measures of relevant alcohol-related outcomes. Both 1) sustained abstinence and also 2) no heavy drinking days are the two potential AUD treatment outcome measures recommended by various bodies [41, 42]. Sustained abstinence is arguably a ‘gold standard’ outcome but is infrequently achieved and reliance on this measure may underestimate treatment effects. Reductions in the World Health Organization (WHO) risk drinking levels [43] have recently been proposed as an alternative primary outcome for all alcohol clinical trials [44] and these endpoints are suitable for primary care alcohol treatment research. Findings among both AUD treatment seekers and the general drinking population show that reductions in WHO risk drinking levels are associated with improvements in physical and mental health such as liver disease, depression and anxiety [45–47]. We suggest consistent reporting of WHO risk levels will facilitate cross comparison of outcomes and also provision of clinically significant measures of improvement as outlined above.

The use of objective markers of alcohol use to corroborate self-report may also serve to improve consistency and quality of alcohol treatment research in primary care settings. One example is phosphatidylethanol (PEth) which is the new gold standard for reliable laboratory corroboration of alcohol consumption [48, 49]. While likely to be less accessible in primary care settings at the current time this may change in future years. Liver enzymes particularly γ-glutamyltransferase (GGT) are highly relevant to harms of AUD and also serve as an objective marker of recent consumption. These tests are readily available in primary care settings and are generally cheap and acceptable to most patients. Falling levels of aspartate transaminase (AST), alanine aminotransferase (ALT), and GGT are strongly correlated with alcohol consumption and associated with better health outcomes [50].

#### Predictors of treatment engagement and response

Patient characteristics such as alcohol severity and readiness to change may potentially predict suitability for alcohol treatment in a primary care setting. The Alcohol Use Disorders identification Test (AUDIT)-C, which is first 3 questions of the 10-item AUDIT, assesses alcohol consumption patterns in the past year and has been validated as a brief alcohol-screening test [51] and widely recommended for use in primary care. While consumption obtained via the AUDIT-C is not always entirely accurate, with potential for underestimation of actual consumption [52], increasing scores are associated with increasing severity of alcohol-related problems in the past 12 months [53].

Patients with higher readiness to change scores are associated with improved treatment engagement and alcohol use outcomes [54]. Thus, the potential for varying degrees of treatment seeking and ambivalence about treatment should be measured given that patients in primary care may not be interested in receiving AUD treatment. There are several validated readiness to change measures such as the Readiness to Change Questionnaire [55] and the Stages of Change Readiness and Treatment Eagerness Scale [56]. However, although relatively brief, these require dedicated data collection and consequently researcher input. Brief assessments and algorithms of readiness to change suitable for primary care also exist with face validity and potentially good concurrent validity when compared with the longer Readiness to Change Questionnaire [57, 58].

#### Method of data collection

The emerging secondary use of electronic medical records (EMRs) for research purposes is occurring throughout the world [59]. As EMRs become more widely adopted in primary health care, research in these settings will be improved. Information from primary care EMRs can be used to evaluate the treatment outcome and uptake and also treatment fidelity, which would be particularly useful for evaluating psychosocial interventions (to the extent that these are recorded). EMRs can also be used to evaluate implementation facilitators and barriers and potentially assist in recruitment by earlier screening for alcohol problems [60]. Data linkage with repositories of primary care clinical data will significantly improve our capacity to evaluate treatment in these settings [61]. While these systems may already be utilised consistently in some countries they are not in many regions. For example, in Australia, there are multiple EMR systems that limit use of primary health care data for research and for data linkage between health care settings [59].

### Limitations

One of the main limitations is the use of varied outcome measures across studies which makes comparison of study findings difficult. In addition, it is important to note that the synthesis of non-inferiority trials, comparing primary care management versus specialty care management, may be complicated by potential varying degrees of treatment seeking in the patients involved and ambivalence about need for treatment. The majority of AUD treatment trials in addiction specialty care settings involve treatment seeking individuals whereas many patients in primary care may often not be interested in receiving treatment for AUD. To this degree, studies examining primary care versus specialty care whereby there was comparable baseline contemplation or previous treatment history, similar or even improved alcohol outcomes were observed in the primary care group [e.g. 20]. This suggests that a null result in non-inferiority trials can be perceived as supporting the recommendation for implementation of AUD treatment into primary care whereby the aim is to facilitate earlier uptake of treatment rather than determining a more effective setting for treatment in comparable patients.

## Conclusion

Models of care in primary care-settings enhanced treatment uptake (psychosocial and/or pharmacotherapy) while the results for alcohol consumption were somewhat mixed. Our findings show that models of care in primary care-settings have promise to be beneficial in the management of AUD in terms of engagement. More studies are required with consistent outcome measures in order to determine effectiveness and cost effectiveness of these models of care, to clarify the most appropriate components of the models and to determine which patients are most suitable.

## Data Availability

Data that support the findings of this study are available from the corresponding author upon reasonable request.

## **Appendix 1**

### Search strategy MEDLINE

Search strategy

- Draft of at least one database

**Table:**

Concept	Description of concept	Research Terms
A	Primary Health Care (PHC)	exp Primary Health Care/ OR exp. General Practice/ OR Primary Care.mp
B	Alcohol Use Disorder	exp Alcoholism/ OR exp. Alcohol Drinking/ OR alcohol dependence.mp. OR alcohol problems.mp. OR hazardous drinking.mp. OR problem drinking.mp. OR AUD.mp
C	Treatment	exp disease management/ OR treatment.mp. OR intervention.mp.
D	Model of care	models of care.mp. OR exp. Delivery of Health Care, Integrated/ OR Patient Care Team/ OR shared care.mp OR Collaborative care.mp OR stepped care.mp. OR multi-faceted care.mp. OR Interdisciplinary treatment approach.mp. OR nurse practitioners/ OR exp. family nurse practitioners/ OR exp. nurse specialists/ OR specialist liaison.mp. OR Chronic Disease/ OR Chronic Care.mp

- Search: A + B + C + D

## **Appendix 2**

### Search strategy grey literature

Grey matters: a practical tool for searching health-related grey literature [internet]. Ottawa, CADTH; 2018.

Terms used: alcohol, primary care, general practice. Most sites had a search engine but almost none allowed for an advanced search.

Grey Matters headings

- Health technology assessment agencies - all searched, some sites were useful
  - International - all searched, some sites were useful (12 useful titles found)
- Health economics - not searched, not relevant
  - International - not searched, not relevant
- Clinical practice guideline - some searched, nothing found
  - International - some searched, some results found (2 useful titles found)
- Drug and Device Regulatory approvals - not searched, not relevant
  - International - not searched, not relevant
- Advisories and warnings - not searched, not relevant
  - International - not searched, not relevant
- Drug Class reviews - not searched, not relevant
- Clinical trial registries - searched, relevant titles found - same studies. 3 protocols and/or conference abstracts of potential useful studies found but no data available yet (emailed authors).
- Canadian drug formularies - not searched, not relevant
- Canadian physician Fees schedules - not searched, not relevant
- Databases - we searched Medline, PsycINFO, Cochrane database of systematic reviews, Cochrane Central Register of Controlled Trials (CENTRAL) and Embase (2019) - as described in the formal text.
- Health statistics - not searched, not relevant
- Search Engine - google and google scholar searched, this yielded a lot of hits. All useful titles were also found through our database search (55 useful titles, no new titles however). As recommended by Grey matters tool - I only went through the first 100 titles. As you cannot do a specific search in google, it yielded > 1500 hits.
- Open access journals - searched, nothing found
- Behavioural change - searched, nothing found
- Natural medicine and environmental health - not searched, not relevant
- Dentistry - not searched, not relevant
- Diagnostic tests - not searched, not relevant
- Mental health - searched, site not working
- Nursing - not searched, not relevant
- Physiotherapy/ rehabilitation - not searched, not relevant

Total hits - unknown.

Useful titles - 72.

Useful titles after removing duplicates from main database search - 17.

Full text read – 14.

Used in systematic review - nil (some of the titles that were found in the google scholar search were used in the systematic review but these articles were also yielded by the database search).

## Abbreviations

AST: Aspartate transaminase
ALT: Alanine aminotransferase
AUD: Alcohol use disorder
AUDIT: Alcohol Use disorders identification test
CBT: Cognitive behavioural therapy
CCM: Chronic care model
CENTRAL: Cochrane Central Register of Controlled Trials
DSM-IV: Diagnostic and statistical manual of mental disorders
EMR: Electronic medical record
GGT: γ-Glutamyltransferase
HDD: Heavy drinks per drinking day
ICD-10: International Statistical Classification of Diseases
IRR: Incidence rate ratios
MET: Motivational enhancement therapy
OAUD: Opioid and alcohol use disorder
OR: Odds ratio
PEth: PhosphatidylEthanol
PRISMA: Preferred reporting items for systematic reviews and meta-analyses
PROSPERO: Prospective register of systematic reviews
RoB: Risk-of-bias
SBIRT: Screening, brief intervention and referral to treatment
SD: Standard drinks
SMD: Standardized mean differences
SUD: Substance use disorder
USA: United States of America
WHO: World Health Organization
